# Cost-effectiveness analysis of ribociclib plus letrozole versus palbociclib plus letrozole in the United Kingdom

**DOI:** 10.36469/9725

**Published:** 2019-04-11

**Authors:** Gaurav Suri, David Chandiwana, Adam Lee, Rohit Mistry

**Affiliations:** 1 PAREXEL International, London, UK; 2 Novartis Pharmaceuticals Corporation, East Hanover, NJ, USA; 3 Novartis UK, London, UK

**Keywords:** Cost-effective, Ribociclib, Palbociclib, HR+/HER2−, advanced breast cancer, UK

## Abstract

**Objective:**

To evaluate the cost-effectiveness of ribociclib plus letrozole versus palbociclib plus letrozole in post-menopausal women with hormone receptor positive (HR+) and human epidermal growth receptor 2 negative (HER2−) advanced breast cancer from a UK payer perspective.

**Methods:**

A cohort-based partitioned survival model was developed to evaluate the cost-effectiveness of ribociclib plus letrozole versus palbociclib plus letrozole in post-menopausal women with HR+/HER2− advanced breast cancer over a lifetime horizon. The analysis was carried out from a National Health Services and Personal Social Services perspective, and results are presented in incremental costs per quality adjusted life years. Clinical data from three randomized controlled trials (MONALEESA-2, PALOMA-1 and PALOMA-2 studies) were used, and supplemented with available real world evidence. Costs categories comprised of drug acquisition, medical management, and treatment of adverse events. Healthcare resource utilization data were identified from literature and unit costs sourced from secondary sources. Utility values were derived from MONALEESA-2 study and were supported with values identified from literature. Both deterministic and probabilistic analyses were carried out to assess uncertainty.

**Results:**

In the base case, treatment with ribociclib plus letrozole increased mean progression free survival (PFS) by 4.1 months and overall survival by 5.0 months compared to palbociclib plus letrozole. Further, treatment with ribociclib plus letrozole resulted in cost-savings of £8464 and incremental QALYs of 0.261, demonstrating that treatment with ribociclib plus letrozole is dominant to treatment with palbociclib plus letrozole. The probabilistic analysis also yielded mean cost-savings of £7914 and mean QALY gain of 0.273. At willingness-to-pay threshold of £30 000 per QALY, treatment with ribociclib plus letrozole had a 92% probability of being cost-effective compared to palbociclib and letrozole.

**Conclusions:**

The results of the analysis demonstrate that ribociclib plus letrozole treatment is both cost-saving and a cost-effective option amongst the available cyclin dependent kinase 4/6 inhibitors for the treatment of post-menopausal women with advanced breast cancer. The biggest driver of the cost savings were the lower acquisition costs of ribociclib.

## Introduction

Globally, breast cancer is the most common cancer in females.^1^ In the United Kingdom (UK), nearly 55 122 new cases were diagnosed in 2015. This represents about 31% of the all the new cancer cases in females.^2^ Approximately 6–7% of patients are diagnosed at late stage (stage IV) and have metastases; where the tumor has spread significantly within the breast or to other organs of the body. These patients tend to have poor prognosis and a dismal one-year survival rate of 63%.^3^,^4^

The mainstay first-line treatment for the estimated 7500 post-menopausal women with advanced breast cancer in the UK are endocrine therapy with steroidal or non-steroidal aromatase inhibitors.^5^ Recent clinical studies have demonstrated the addition of cyclin-dependent kinase 4 and 6 inhibitors (CDK 4/6) inhibitors (such as ribociclib and palbociclib) to aromatase inhibitors (such as letrozole) provides rapid clinical improvement in patients with measurable disease. With recent Food and Drug Administration (FDA) and European Medicines Agency (EMA) approval of CDK 4/6 inhibitors treatment landscape for post-menopausal women with hormone receptor positive (HR+) and human epidermal growth receptor 2 negative (HER2−) advanced breast cancer is likely to change and is going to bring about new alternatives.

Both ribociclib and palbociclib are administered orally (licensed dose: ribociclib- 600 mg, palbociclib- 125 mg; dose reduction due to adverse events or intolerance: ribociclib- 400/200 mg, palbociclib- 100/75 mg) in a 3-weeks on/1-week off schedule in combination with letrozole (2.5 mg once daily). These CDK inhibitors possess similar mechanism of action and have demonstrated clinical efficacy in separate randomized trials.^6^–^8^

Clinical efficacy of ribociclib plus letrozole was assessed in the MONALEESA-2 study.^6^ The MONALEESA-2 (NCT01958021) was a randomized, double-blinded, placebo controlled Phase III study that compared ribociclib plus letrozole and placebo plus letrozole. Post-menopausal women with HR+/HER2− advanced breast cancer who received no prior therapy for advanced disease, were randomly assigned to either ribociclib (600 mg daily, 3-weeks on/1-week off in a 4-week cycle) plus letrozole (2.5 mg once daily) or placebo (once daily, 3-weeks on/1-week off in a 4-week cycle) plus letrozole (2.5 mg once daily). The primary endpoint was progression-free survival (PFS); defined as time from the date of randomization to the date of the first documented progression or death due to any cause, as determined by investigator (via RECIST 1.1 criteria). The study results demonstrated statistical significant benefit observed in subjects who received ribociclib plus letrozole over placebo plus letrozole in PFS (HR = 0.568, p-value =3.29 × 10^−6^).

Efficacy data for palbociclib plus letrozole was derived from two PALOMA studies [PALOMA-1 (NCT 00721509) & PALOMA-2 (NCT01740427)]. Both were multi-center randomized studies and compared palbociclib plus letrozole and placebo plus letrozole; whilst PALOMA-1 was a Phase II open label study, PALOMA-2 was double-blinded Phase III study. Across both studies, post-menopausal women with estrogen receptor positive HER2− advanced breast cancer who have not received prior systematic treatment were randomized to either palbociclib (125 mg once daily, 3-weeks on/1-week off in a 4-week cycle) plus letrozole (2.5 mg once daily) or placebo (once daily, 3-weeks on/1-week off in a 4-week cycle) plus letrozole (2.5 mg once daily). Results in both studies confirmed statistical significant improvement in primary endpoint (PALOMA-1: HR = 0.488, p-value = 0.004; PALOMA-2: HR = 0.58, p-value <0.001). Further details of these analyses are presented elsewhere.^7^,^8^

Earlier evaluations have estimated cost-effectiveness of different endocrine therapies and conventional aromatase inhibitors in first-line treatment of post-menopausal women.^9^–^12^ But no study has yet carried out a cost-effectiveness comparison of two CDK inhibitors in post-menopausal women with advanced breast cancer in the UK. This study aims to fill that gap, and evaluate the cost-effectiveness of ribociclib plus letrozole versus palbociclib plus letrozole, to inform the decision makers.

## Methods

A de-novo cohort based partitioned survival model was developed in Microsoft Excel® to estimate the cost-effectiveness of ribociclib plus letrozole versus palbociclib plus letrozole. This analysis evaluated the expected costs, life-years and quality-adjusted life years (QALYs) using a 4-week cycle for a life time horizon of 40 years. The cohort characteristics modelled in the analysis were based on the patients enrolled in the MONALEESA-2 study which was similar to those enrolled in the two PALOMA trials.

The analysis was carried out from a UK National Health Services (NHS) and Personal Social Services (PSS) perspective and included all direct medical costs pertinent to the NHS, which included costs pertaining to drug acquisition and monitoring, health state specific disease monitoring, subsequent therapy and management of adverse events costs. The effectiveness (benefits) of the treatment was evaluated using a generic measure for disease burden via QALYs. The cost-effectiveness results were expressed in incremental cost-effectiveness ratios (estimated as a ratio of incremental costs to incremental QALYs).

### Structure

**Figure 1:**
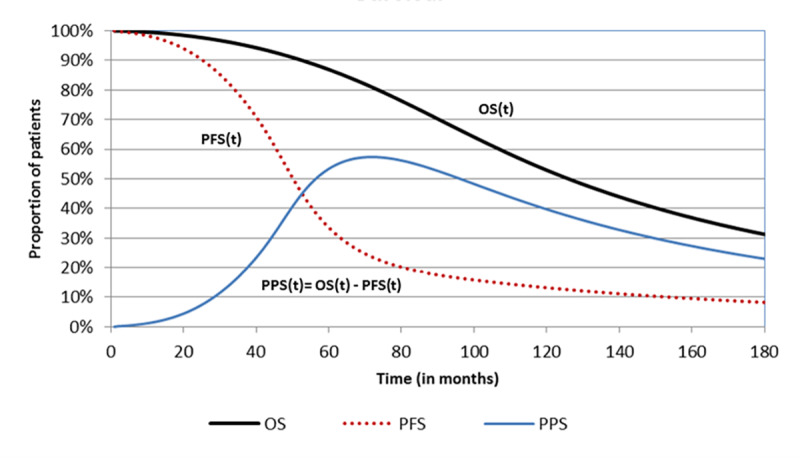
Survival curves based on PFS and OS data to track state occupancy PFS: progression-free survival; PPS: post-progression survival; OS: overall survival The proportion of patients in the progressed-disease state are presented in the figure under the post-progression curve

A three-health state structure including progression-free, progressed-disease and death was used to track patients along the disease pathway (Figure 1). Time-dependent state occupancy for each health state in the model was estimated from survival, modelled using PFS and overall survival (OS) data from MONALEESA-2 study. The proportion of patients alive who have not progressed (occupying progression-free state) were estimated using PFS data. Patients in the PF state were sub-divided patients into those who achieved objective response to the treatment, and those who remained progression-free with stable disease. This categorization of patients was carried out to account for the health-effects of tumor reduction. The proportion of dead patients (occupying the death state) was estimated from OS data. The proportion of patients occupying the progressed-disease state at each interval were estimated as the difference of the alive patients (estimated from the OS data) and the proportion of patients who haven’t progressed (estimated from the PFS data).

### Clinical Parameters

Survival for ribociclib plus letrozole and palbociclib plus letrozole were derived by applying a hazard ratio (versus placebo plus letrozole) to the reference arm modelled by fitting parametric functions to PFS and OS data for placebo plus letrozole in the MONALEESA-2. The hazard ratio for ribociclib plus letrozole (vs. placebo plus letrozole) was derived using a matched-adjusted indirect comparison (MAIC).13 The MAIC adjusted for the small differences in patient characteristics across the studies (i.e. PFS: MONALEESA-2 and PALOMA-2 and OS: MONALEESA-2 and PALOMA-1). Further details of the MAIC are presented elsewhere. For palbociclib and letrozole (vs. placebo plus letrozole) hazard ratios were derived using a conventional indirect treatment comparison.^13^

The selection of best fitting parametric function was based on statistical goodness of fit indicators as well as clinical plausibility as recommended in NICE DSU document on survival analysis modelling.^14^ Despite a higher Akaike information criteria (AIC) value, PFS for placebo plus letrozole was modelled using exponential distribution. This was done as the 5- and 10-year landmark projections fell within the range recommended by the clinical expert. The OS for placebo plus letrozole was modelled using Weibull distribution.

The proportion of patients in the PF sub-states was modelled with treatment specific overall response rate. Treatment specific response rates for ribociclib plus letrozole and palbociclib plus letrozole were derived by applying odds ratio to the rates observed in the placebo plus letrozole arm of MONALEESA-2 study. The response rates were assumed to follow a linear trend for the first 12 months in line with clinical data. Beyond month 12, proportion of responders was held stable but the number of responders was assumed to decline with the PFS.

### Costs

The drug acquisition cost of each therapy were based on pack prices sourced from British National Formulary.^15^ The healthcare resource use pertaining to drug monitoring was identified from summary of product characteristics presented in respective FDA drug labels. The unit costs for resource use were sourced from publicly available tariffs in the UK,^16^ and were inflated to 2016 where necessary.

The total drug acquisition costs in the model were based on time to treatment discontinuation. Consistent with PFS and OS, duration of treatment for ribociclib plus letrozole was modelled by fitting a parametric function (exponential) model to time to treatment discontinuation data for ribociclib and letrozole from MONALEESA-2 study. For palbociclib plus letrozole, this was modelled by applying hazard ratio for PFS (comparing ribociclib plus letrozole vs. palbociclib plus letrozole) obtained from the meta-analysis to the parametric model fitted to ribociclib plus letrozole data (Table 1).

The model adjusted for the dose reduction (from 600 mg to 400 mg, and 400 mg to 200 mg) for ribociclib based on clinical data (for the first 16 months) from MONALEESA-2 study. Dose-wise distribution of patients beyond 16 months was assumed same as that observed at 16 months. For palbociclib (which allowed dose reduction from 125 mg to 100 mg, and 100 mg to 75 mg), any dose reduction for palbociclib was likely to result in drug wastage. This was based on data from a real world study that evaluated the utilization pattern of palbociclib by analyzing prescription claims database in the United States.^17^

Disease monitoring healthcare resource use data was informed by literature. The health-state specific resource use included costs for healthcare professional visits, hospitalization, monitoring and imaging. Total monthly costs for each of the healthcare resource use were estimated as a product of proportion of patients accruing these costs with the resource use and its specific unit costs.

Healthcare professional visits included costs associated with visits to the general practitioner, oncology consultant, community nurse, clinical nurse specialist, social worker or radiographer. Hospitalization costs accounted for general and oncology specific and were applied to a proportion of patients. Routine monitoring costs included costs related to blood tests. Imaging costs comprised of bone-scintigraphy costs, x-rays, ultrasound, magnetic resonance imaging and positron emission tomography. Further details of these cost elements are presented in Table 1.

A monthly subsequent therapy cost was also applied to account for the costs of further therapies (either endocrine or chemotherapy) in the second and third line following disease progression using the same methodology as used by Das and colleagues.^12^ This cost was assumed the same irrespective of the treatment received prior to progression. Firstly, total costs were estimated by apportioning the progressed patients into those who received further treatment in a) second and b) third line setting. The proportional split of patients into endocrine and chemotherapy across both lines were based on utilization rates of case mix of treatment therapies.^18^,^19^ These total costs were then divided by a post-progression survival of 20.5 months,^20^ to estimate a monthly cost which was applied to all the patients after progression.

### Quality of Life

The MONALEESA-2 study collected health-related quality of life data using EuroQoL 5-dimension (EQ-5D-5L) questionnaire during the screening phase, with follow-up every eight weeks during the first 18 months and then every 12 weeks thereafter until disease progression or at the end of treatment. Health-state utility values were estimated using EQ-5D-5L UK social tariff reported by Devlin *et al*.^21^ Separate values were generated for responders (complete or partial) and those who were in progression-free and had stable disease. Values for the progressed disease state was based on a study that reported values in patients with metastatic breast cancer.^22^ The values for the progressed disease were based on literature as the PD values in the trial were assessed for a follow-up duration of only 30 days beyond end of treatment. A summary of the health-state utility values used in the model is presented in Table 1.

### Sensitivity Analysis

Both one-way deterministic and probabilistic analyses were performed to adjust for the uncertainty associated with the key parameters. In the one-way sensitivity analysis, the values of parameters were varied by its 95% CI (where available, otherwise assumed ±10%). The results of one-way sensitivity analyses were presented in Tornado plot depicting the impact on the net monetary benefit for the parameters that had the biggest impact.

A probabilistic analysis using a Monte Carlo simulation of 1000 iterations was performed.^23^,^24^ The simulation varied input parameters by sampling using appropriate distributions. The input parameters sampled in the probabilistic sensitivity analysis included clinical parameters such as hazard ratios, odds ratios, incidence rates of adverse events, and quality of life inputs such as health state utility values. The results of the probabilistic sensitivity analyses were plotted on a cost-effectiveness plane, and cost-effectiveness acceptability curve.

An additional scenario analysis was also carried out that used an alternative distribution (Weibull) for modelling the placebo plus letrozole PFS to which the HR’s were applied. This distribution was selected in the scenario analysis as it has been used to model PFS in women with HR+/HER2− advanced breast cancer in an earlier economic evaluation.^25^ This analysis was carried out to assess the impact of using another plausible distribution for modelling the PFS; all other model settings were kept similar to the base-case.

## Results

Compared to palbociclib plus letrozole, ribociclib plus letrozole treatment increased mean PFS by 4.1 months and mean OS by 5.0 months over a 40-year time horizon. Treatment with ribociclib plus letrozole was cheaper and resulted in a greater QALYs compared to palbociclib plus letrozole. The total treatment costs with ribociclib plus letrozole and palbociclib plus letrozole were £104 230 and £112 694. The total QALYs were 3.296 and 3.034. This resulted in the dominance of ribociclib plus letrozole over palbociclib and letrozole. A breakdown of the costs and health benefits is presented in Table 2. The majority of cost savings were because of the savings made in the acquisition costs of the therapies.

**Figure 2:**
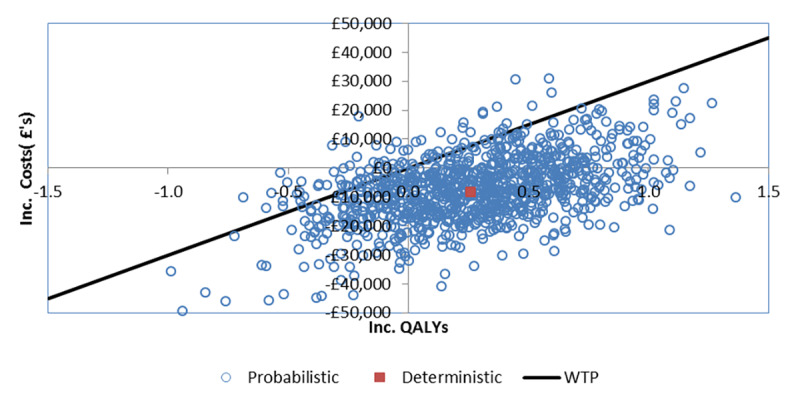
Cost-effectiveness plane WTP: Willingness to pay threshold at £30 000/QALY QALY: Quality-adjusted life year

**Figure 3:**
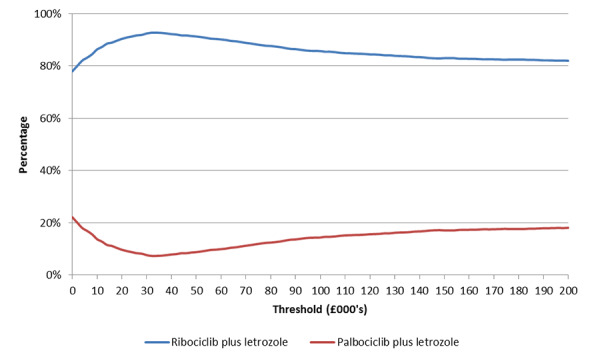
Cost-efficiency acceptability curve

The probabilistic analyses also confirmed the results of the deterministic analyses. Based on a mean costs savings of £7914 (95% CI: £7868, £7960) and mean incremental QALYs of 0.273 (95% CI: 0.273, 0.274) treatment with ribociclib was cost-effective compared to palbociclib in 92% of the probabilistic simulations (at a willingness to pay threshold of £30 000, more than 57% of the iterations demonstrated dominance of ribociclib plus letrozole treatment over palbociclib plus letrozole), and was dominated in 1.3% of the iterations. The results of the probabilistic analyses are presented in Table 2. The cost-effectiveness plane and the cost-efficiency acceptability curve are presented in Figure 2 and Figure 3 respectively. At thresholds of £20 000 and £50 000 the probabilities of ribociclib plus letrozole being cost-effective was 90% and 91% respectively.

**Figure 4:**
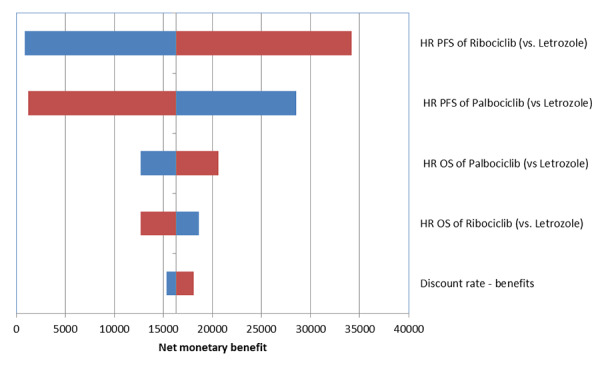
Tornado plot demonstrating the top five key drivers of cost-effectiveness results HR: Hazard ratio; OS: Overall survival; PFS: Progression-free survival; vs.: versus

Results of the one-way deterministic sensitivity analysis are presented in the Tornado plot (Figure 4). Key model drivers based on the change in the net monetary benefit (rather than the more conventionally used ICERs was used for easier interpretation of scenarios with cost-savings and incremental QALYs comparing ribociclib plus letrozole and palbociclib plus letrozole) were the PFS HR for ribociclib plus letrozole versus letrozole, PFS HR for palbociclib plus letrozole versus letrozole, OS HR for ribociclib plus letrozole versus letrozole, OS HR for palbociclib plus letrozole versus letrozole and the discounting rate for benefits.

The scenario analysis using the Weibull distribution also demonstrated cost savings with ribociclib plus letrozole treatment. The savings were £7884 compared to the base-case analysis.

## Discussion

Breast cancer is a life-threatening form of cancer; having severe impact on survival in patients with this condition. This necessitates urgent need for therapies that have better impact on reducing the risk of progression or death in patients with HR+/HER2− advanced breast cancer.

EMA granted marketing authorization for use of ribociclib for the treatment of locally advanced or metastatic breast cancer.^26^ The approval was based on the results of MONALEESA-2 study which demonstrated significant improvement in PFS despite an immature OS. The assessment report indicated that OS results (at an event rate of 17% at the time) were supportive of PFS, and noted a trend towards OS benefit.^27^

This is the first study that evaluated the cost-effectiveness of ribociclib plus letrozole versus palbociclib and letrozole in post-menopausal women with HR+/HER2− advanced breast cancer from a UK payer perspective. Clinical data were derived from MONALEESA-2, PALOMA-1 and PALOMA-2 studies. The study results demonstrated slight clinical benefit (increased PFS and OS) with ribociclib treatment compared to palbociclib treatment. Consequently, ribociclib plus letrozole treatment was associated with an incremental QALY gain of 0.261 over lifetime. The results also indicate cost savings with ribociclib treatment. Unlike ribociclib, dose reduction for palbociclib results in potential drug wastage that has the cost implications and was one of the drivers of the cost savings. These cost savings were due to the linear pack pricing (cost per mg) for ribociclib compared to flat pack pricing for palbociclib; with patients moving to the lower (therefore less costly) ribociclib doses over time.

There are some limitations of the analysis. Firstly, there is uncertainty regarding the parametric survival modelling and extrapolation of the OS endpoint. As both ribociclib and palbociclib are recently launched CDK inhibitors, there are no studies which have presented longer term survival for patients who received these. Therefore in order to validate, we compared the OS projections for placebo and letrozole which was used for modelling survival of ribociclib plus letrozole and palbociclib plus letrozole. The predicted survival projections for placebo and letrozole (using exponential distribution) of 95%, 82% and 26% at 1-, 2- and 5- year landmark closely aligned the OS projections elicited through expert consultation and literature.^28^,^29^ Given the uncertainty around the extent of impact of CDK inhibitors on the OS, the assessment warrants further analysis in future when mature OS data become available.

The second limitation is that the key inputs in the analysis such as utilities and time on treatment were assumed to be equal between the two treatments. This was due in part to lack of publicly available data from the PALOMA-2 study. Given the dosing structure of the two drugs, it is highly likely that time on treatment might be different and therefore might have an impact on the results.

Furthermore, the analysis evaluated cost-effectiveness of ribociclib plus letrozole vs. palbociclib and letrozole using effect estimates derived from a MAIC in the absence of direct comparative evidence. There may be some uncertainty in this indirect analysis compared with head to head evidence from a direct comparative study (i.e. comparing ribociclib plus letrozole vs. palbociclib plus letrozole).

Recently, preliminary results for abemaciclib in combination with fulvestrant for the treatment of postmenopausal women with HR+/HER2− advanced breast cancer based on phase III study MONARCH-3 are published.^30^ However, abemaciclib had not received FDA or EMA approval at the point of model development, and therefore was not included in the study.^31^ Given the approval of abemaciclib in the same patient population would require a fully incremental analysis of the cost-effectiveness of all the CDK4/6 inhibitors.

## Conclusions

The results of this study demonstrate that combination therapy of ribociclib and letrozole for the treatment of post-menopausal women with HR+/HER2− advanced breast cancer would be a cost-effective option compared to palbociclib and letrozole from a NHS and PSS perspective in the UK. The biggest driver for the cost-effectiveness results were the lower drug acquisition costs for ribociclib. Additional data from both the MONALEESA-2 and PALOMA-2 studies will increase the robustness of the cost-effectiveness estimates.

## Figures and Tables

**Table 1:** Model inputs

Characteristics	Ribociclib plus letrozole	Palbociclib plus letrozole
**Clinical efficacy**		
Rate parameter for PFS^a^	0.045	
Shape parameter for OS^b^	2.110	
Scale parameter for OS^b^	52.278	
Overall response rate^c^	28.74%	
Rate parameter for TTD^d^	0.042	
Hazard ratio for PFS	0.524 (0.407, 0.676)	0.580 (0.460, 0.720)
Hazard ratio for TTD	-	1.000 (0.730, 1.390)
Hazard ratio for OS	0.682 (0.456, 1.021)	0.813 (0.492, 1.345)
Odds ratio for OR^e^	1.420 (1.200, 1.66)	1.230 (1.030, 1.440)
**Costs (mean monthly)**		
Drug acquisition	£2 950/£1 967/£983	£2 950
**Healthcare resource use**^f^	**PF**	**PD**
Healthcare professional visits	£255	£302
Hospitalization	£147	£1 031
Monitoring	£1	£3
Imaging	£41	£49
Total	£445	£1 384
**Subsequent therapy costs**^g^		
Second line (endocrine)	£6579	
Second line (chemotherapy)	£1285	
Third line (endocrine)	£7499	
Third line (chemotherapy)	£1716	
Total cost of subsequent therapies	£5823	
Monthly subsequent therapy	£284	
**Utility values**		
Progression-free (response)	0.8345 (0.0068)	
Progression-free (SD)	0.8296 (0.0063)	
Progressed-disease	0.5050 (0.0443)	

a Parameters for parametric model via exponential distribution (with AIC values = 1693.3) fitted to placebo plus letrozole arm for OS endpoint to which the HR (vs. placebo plus letrozole) for ribociclib plus letrozole and palbociclib plus letrozole were applied

b Parameters for parametric model via Weibull distribution (with AIC values = 733.7) fitted to placebo plus letrozole arm for OS endpoint to which the HR (vs. placebo plus letrozole) for ribociclib plus letrozole and palbociclib plus letrozole were applied

c Response rates observed in the placebo plus letrozole

d Parameter for parametric model via exponential distribution (with AIC values = 1837.6) fitted to ribociclib plus letrozole arm for TTD endpoint to which the HR (vs. palbociclib plus letrozole) were applied

e Odds ratios for overall response (relative to placebo plus letrozole) were applied to proportion of patients responding to the placebo plus letrozole treatment to generate the response rate in the ribociclib and palbociclib arm

f Health care resource use data were assumed conditional on health state irrespective of the combination therapy

g Subsequent therapy costs were also assumed similar for both combination therapy

OR: Overall response, OS: Overall survival, PD: Progressed-disease, PF: Progression-free, PFS: Progression–free survival, TTD: Time-to-treatment discontinuation

**Table 2:** Results of the cost-effectiveness analysis

Treatment	Clinical projections	Costs	Total QALYs	Inc. costs	Inc. QALYs	ICER (Inc. costs/Inc. QALYs)
**Deterministic**						

	Modelled PFS at					
	1 year: 74.6%	Total: £104 230				
	2 years: 56.3%	Acquisition: £58 358				
	5 years: 24.2%	Drug monitoring: £602				
	40 years: 0.0%	Drug-wastage: £0	Total: 3.296			
Ribociclib		PFS costs: £17 179	PF: 2.673			
	Modelled OS at	PD costs: £20 466	PD: 0.68			
	1 year: 96.7%	Subs. therapy costs: £4201		Total: −£8464		
	2 years: 87.1%	Terminal care: £3266		Acquisition: −£10 166		
	5 years: 39.5%	AE: £157		Drug monitoring: £525		
	40 years: 0.0%			Drug-wastage: −£1482	Total: 0.261	
			
				PFS costs: £1495	PFS: 0.233	Ribociclib is dominant
	Modelled PFS at			PD costs: £1107	PD: 0.028	
	1 year: 72.3%	Total: £112 694		Subs. therapy costs: £191		
	2 years: 52.9%	Acquisition: £68 524		Terminal care: −£44		
	5 years: 20.7%	Drug monitoring: £77		AE: £85		
	40 years: 0.0%	Drug-wastage: £1482	Total: 3.034			
Palbociclib		PFS costs: £15 684	PF: 2.673			
	Modelled OS at	PD costs: £19 535	PD: 0.68			
	1 year: 96.1%	Subs. therapy costs: £4010				
	2 years: 84.8%	Terminal care: £3310				
	5 years: 33.1%	AE: £72				
	40 years: 0.0%					

**Probabilistic**						

Ribociclib	NE	£107 740 (95% CI: £107 265, £108 214)	3.404 (95% CI: 3.389, 3.418)	−£7914	0.273	Ribociclib is dominant

Palbociclib	NE	£115 654 (95% CI: £115 134, £116 173)	3.130 (95% CI: 3.116, 3.144)			

AE: Adverse event, 95% CI: 95% confidence interval, ICER: Incremental cost-effectiveness ratio, NE: Not evaluated, PD: Progressed-disease, PF: Progression-free, PFS: Progression-free survival, QALYs: Quality-adjusted life years
